# PAM staining intensity of primary neuroendocrine neoplasms is a potential prognostic biomarker

**DOI:** 10.1038/s41598-020-68071-6

**Published:** 2020-07-02

**Authors:** Timothy M. Horton, Vandana Sundaram, Christine Hye-Jin Lee, Kathleen Hornbacker, Aidan Van Vleck, Kaisha N. Benjamin, Allison Zemek, Teri A. Longacre, Pamela L. Kunz, Justin P. Annes

**Affiliations:** 1 Department of Chemistry, Stanford University, Stanford, CA USA; 2 Chemistry, Engineering and Medicine for Human Health (ChEM-H) Institute, Stanford University, Stanford, CA USA; 3 Quantitative Sciences Unit, Department of Medicine, Stanford University, Stanford, CA USA; 4 Division of Endocrinology, Department of Medicine, Stanford University, CCSR 2255-A, 1291 Welch Rd., Stanford, CA 94305-5165 USA; 5 Endocrine Oncology Program, Stanford University, Stanford, USA; 6 Division of Oncology, Department of Medicine, Stanford University, Stanford, CA USA; 7 Department of Bioengineering, Stanford University, Stanford, CA USA; 8 Department of Pathology, Stanford University, Stanford, CA USA

**Keywords:** Immunohistochemistry, Neuroendocrine cancer, Tumour biomarkers, Prognostic markers

## Abstract

Neuroendocrine neoplasms (NENs) are rare epithelial tumors with heterogeneous and frequently unpredictable clinical behavior. Available biomarkers are insufficient to guide individual patient prognosis or therapy selection. Peptidylglycine α-amidating monooxygenase (PAM) is an enzyme expressed by neuroendocrine cells that participates in hormone maturation. The objective of this study was to assess the distribution, clinical associations and survival implications of PAM immunoreactivity in primary NENs. Of 109 primary NENs, 7% were PAM-negative, 25% were PAM-low and 68% were PAM-high. Staining intensity was high in small bowel (p = 0.04) and low in stomach (p = 0.004) NENs. PAM staining was lower in higher grade tumors (p < 0.001) and patients who died (p < 0.001) but did not vary by tumor size or stage at surgery. In patients who died, time to death was shorter in patients with reduced PAM immunoreactivity: median times to death were 11.3 (PAM-negative), 29.4 (PAM-low) and 61.7 (PAM-high) months. Lower PAM staining was associated with increased risk of death after adjusting for disease stage [PAM negative, HR = 13.8 (CI: 4.2–45.5)]. PAM immunoreactivity in primary NENs is readily assessable and a potentially useful stage-independent predictor of survival.

## Introduction

Neuroendocrine neoplasms (NENs) arise from epithelial cells of the neuroendocrine system located throughout the body, most commonly occurring in the gastrointestinal tract, pancreas and lung^1^. Although NENs are considered to be rare, their incidence in the United States is rising rapidly, with a 6.4-fold increased age-adjusted incidence from 1973 (1.09 per 100,000 persons) to 2012 (6.98 per 100,000 persons)^2^. In fact, the generally slow progression of NENs results in a prevalence that exceeds the combined prevalence of multiple gastrointestinal cancers including esophageal cancer, gastric adenocarcinoma and pancreatic adenocarcinoma^3^. Hence, the clinical importance of NENs, historically underappreciated, is gaining recognition^4^.

Given the heterogeneity of anatomic location, histologic appearance and clinical behavior of NENs, establishing consistent nomenclature and pathologic classification criteria has been a challenge^5^. To pathologically identify a NEN, Chromogranin A (CgA) and synaptophysin are considered the most specific immunohistochemical (IHC) markers and are generally required for diagnosis; however, other tumors may stain focally for these markers and exhibit neuroendocrine features^6^. Presently, endocrine tumors are often classified using the World Health Organization (WHO) criteria^7^ which include anatomic location, histologic appearance (well- or poorly-differentiated), WHO grade (Grade 1–3, based upon Ki-67 proliferation index and/or mitotic count) and stage (TNM). Additionally, NENs may be classified as functional if they secrete a peptide hormone associated with symptoms (e.g. insulin and hyperinsulinemic hypoglycemia) or bioactive amines (e.g. serotonin and carcinoid syndrome). While useful prognostic information is provided by the site of origin, degree of differentiation, WHO grade, stage, presence of necrosis and microscopic invasiveness^1,2,8,9^, predicting the behavior of individual well-differentiated NEN remains a challenge. Some NENs grow slowly or do not recur after resection, while others behave aggressively and rapidly advance^10,11^.

Recently, several investigators have sought to identify improved NEN prognostic biomarkers. Genomic sequencing of NENs has identified a variety of somatic mutations that may influence prognosis^12–14^. For instance, early stage pancreatic NENs (panNENs) harboring mutations in TSC2, KRAS or TP53 are associated with reduced survival duration^13^. Similarly, loss of DAXX/ATRX immunostaining has also emerged as a potential poor prognostic indicator for panNENs, though results have been variable^15–17^. Finally, overexpression of somatostatin receptors 2a and 5 (SSTR2a and SSTR5) by well-differentiated NENs predicts longer and progression-free survival^18–20^. Given the numerous factors that influence NEN behavior, attempts have been made to generate predictive nomograms that incorporate several biomarkers and guide individual assessment^10,21^. Unfortunately, these nomograms are not sufficiently reliable for widespread adaptation into clinical practice. Rectifying the current lack of reliable tumor markers for predicting NEN metastatic potential, prognosis and treatment responsiveness remains a challenge for the field^22^. In particular, improved biomarkers which, similar to Ki-67, demonstrate utility across all primary NEN sites of origin, are of highest utility and interest.

Peptidylglycine α-amidating monooxygenase (PAM) is an oxygen-, ascorbate- and copper-dependent enzyme that is expressed in healthy neuroendocrine cells where it plays a necessary role in the maturation of numerous secreted peptide hormones and chromogranin A^23–26^. Several decades-old studies identified PAM expression in NENs of the pancreas, intestine, pituitary, adrenal medulla, medullary thyroid and lung^27–31^; however, the frequency and extent of PAM expression in NENs, and the relationship with tumor prognosis are unknown. Interestingly, reduced PAM expression, based upon oligonucleotide hybridization (immunohistochemical staining was not assessed), was previously associated with malignant behavior in pheochromocytomas^32^. Given the role of PAM in normal neuroendocrine cell function, loss of PAM expression could represent an early indication of NEN de-differentiation, which conveys a poor prognosis^33^. The objective of our study was to investigate the frequency and intensity of PAM immunohistochemical reactivity in a series of primary NENs and explore whether PAM expression was associated with tumor characteristics or patient survival.

## Results

### PAM is present in most primary NENs but levels vary by location

**Figure 1:**
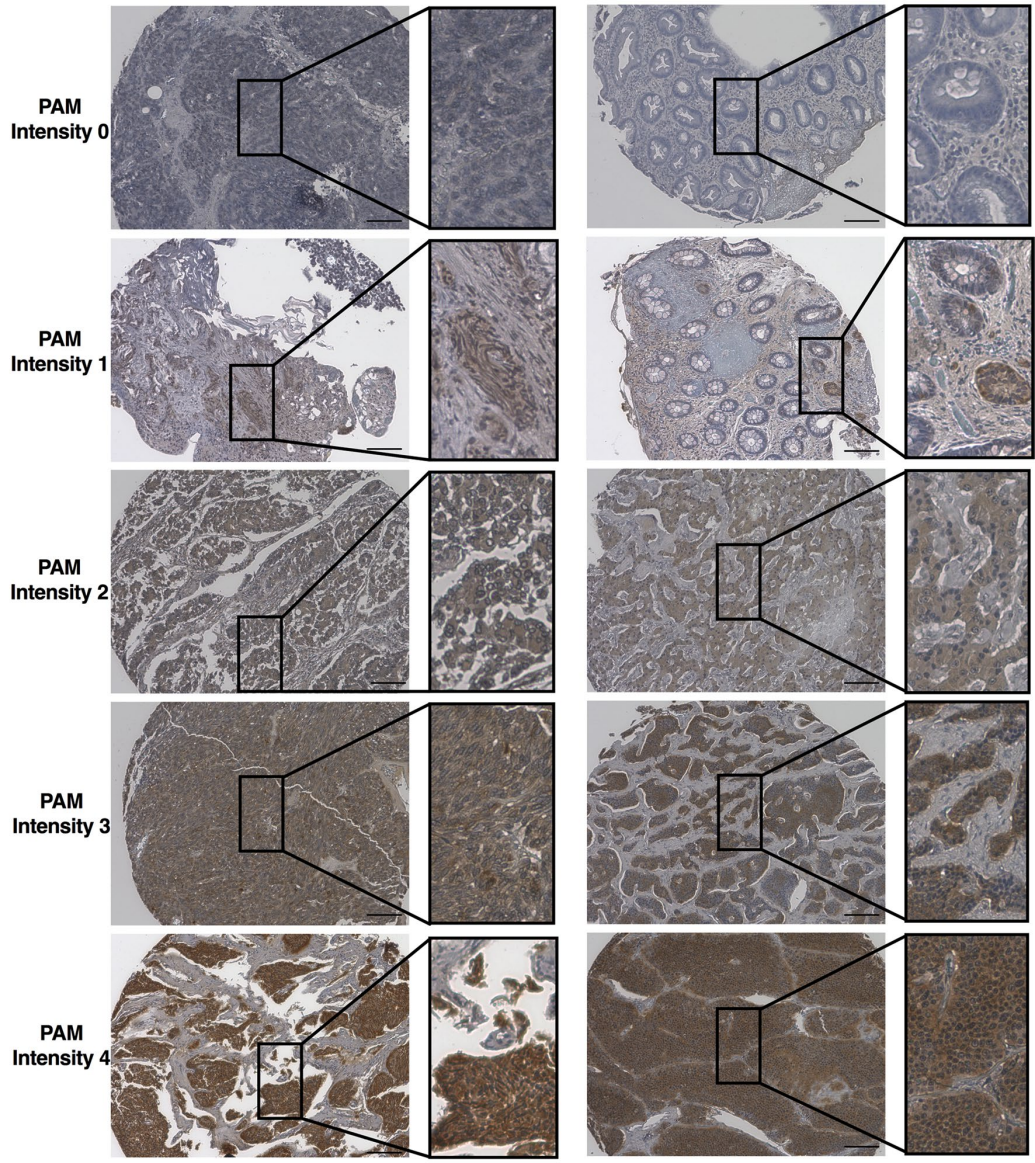
Primary neuroendocrine neoplasms variably express peptidylglycine α-amidating monooxygenase (PAM). PAM-directed immunohistochemistry was analyzed from 109 primary neuroendocrine neoplasms included in a neuroendocrine tumor tissue microarray (described in “Methods” section). PAM staining intensity was scored from zero (no reactivity) to four (strong reactivity). Representative staining and scoring are shown (bar = 200 µm).

We assessed the intensity of PAM expression in a cohort of 109 primary NENs (Table 1, Supplementary Table S1). Tumors were obtained from 62 women and 47 men. Representative images of PAM immunoreactivity are shown in Fig. 1. The median PAM score determined from three investigators was used for analysis. We observed a high degree of agreement between independent reviewers (Krippendorff’s alpha = 0.85 (95% CI: 0.80–0.88); Supplementary Table S1), indicating robust consistency in the assessment of PAM immunoreactivity. Among 109 tumors, 8 were PAM-negative (score = 0), 27 were PAM-low (score = 1) and 74 were PAM-high (score 2–4) (Table 1). Overall, PAM staining intensity of NENs did not significantly vary by anatomic site (Table 1, p = 0.06); however, high PAM-staining was observed more frequently in NENs of the small bowel whereas low PAM-staining was more frequent in NENs of the stomach (Table 2). PAM staining intensity was not associated with patient sex or tumor functional status.

**Table 1:** Characteristics of study population by PAM expression group.

**Characteristic**	**PAM expression group**						**All patients**		**p-value**^**a**^
	**Negative (0)**		**Low (1)**		**High (2 to 4)**				
Total	8 (7%)		27 (25%)		74 (68%)		109		
Age at diagnosis, years; Median (IQR)	66 (60–70)		61 (54–73)		62 (50–70)		61 (52–70)		0.46
Sex, n (%)									0.41
F	5	62.5	18	66.7	39	52.7	62	56.9	
M	3	37.5	9	33.3	35	47.3	47	43.1	
Site, n (%)									0.06
Lung	4	50.0	5	18.5	21	28.4	30	27.5	
Pancreas	0		6	22.2	18	24.3	24	22.0	
Small bowel	0		5	18.5	19	25.7	24	22.0	
Large bowel	2	25.0	6	22.2	11	14.9	19	17.4	
Stomach	0		4	14.8	3	4.1	7	6.4	
Other ^b^	2	25.0	1	3.7	2	2.7	5	4.6	
Functional status, n (%)									0.58^c^
Functional	0		3	11.1	15	20.3	18	16.5	
Non-functional	5	62.5	17	63.0	49	66.2	71	65.1	
Unknown	3	37.5	7	25.9	10	13.5	20	18.4	
WHO Grade									< 0.001^c^
1 (Ki67 < 3)	1	12.5	12	44.4	57	77.0	70	64.2	
2 (Ki67 3 to 20)	0		3	11.1	5	6.8	8	7.3	
3 (Ki67 > 20)	5	62.5	7	25.9	1	1.4	13	11.9	
Unknown	2	25.0	5	18.5	11	14.9	18	16.5	
Tumor size (cm, mean (SD))	3.1 (0.8); n = 3		2.8 (2.0); n = 18		2.2 (2.1); n = 60		2.3 (2.0); n = 81		0.09
Stage at resection, n (%)									0.09^c^
Unknown	2	25.0	4	14.8	9	12.2	15	13.7	
1	2	25.0	8	29.6	30	40.5	40	36.7	
2	0		3	11.1	14	18.9	17	15.6	
3	3	37.5	2	7.4	9	12.2	14	12.8	
4	1	12.5	10	37.0	12	16.2	23	21.1	
Died	8	100.0	15	55.6	15	20.3	38	34.9	< 0.001
Time to death (months); Median (IQR)	11.3 (2.4–23.1)		29.4 (4.1–76.2)		61.7 (41.8–124.2)		48 (11.6–80.7)		

^a^Based Fisher’s exact or Kruskall–Wallis test; IQR: interquartile range; NE: not evaluable.

^b^Other sites include the mediastinum, mesentery, bladder, ovary, and uterus.

^c^After excluding unknown category.

**Table 2:** PAM staining intensity according to the anatomic location of the primary NET.

**Tumor site**	**PAM expression group**				**Mean (SD) staining intensity**	**p-value**^**a**^
	**Negative (0) 8 (7%)**	**Low (1) 52 (48%)**	**High (2 to 4) 49 (45%)**	**All Patients 109**		
Total					2.2 (1.2)	
Lung	4 (13.3)	13 (43.3)	13 (43.3)	30 (27.5)	2.1 (1.3)	0.64
Lobe	4 (14.3)	11 (39.3)	13 (46.4)	28 (25.7)		
Bronchus	0	2 (100.0)	0	2 (1.8)		
Small bowel	0	9 (37.5)	15 (62.5)	24 (22.0)	2.7 (1.1)	0.04
Duodenal	0	3 (60.0)	2 (40.0)	5 (4.6)		
Jejunum	0	1 (100.0)	0	1 (0.9)		
Ileum	0	3 (23.1)	10 (76.9)	13 (11.9)		
Pancreas	0	10 (41.7)	14 (58.3)	24 (22.0)	2.5 (1.1)	0.14
Large bowel	2 (10.5)	11 (57.9)	6 (31.6)	19 (17.4)	1.9 (1.2)	0.20
Colon	2 (25.0)	5 (62.5)	1 (12.5)	8 (7.3)		
Appendix	0	2 (66.7)	1 (33.3)	3 (2.8)		
Rectal	0	4 (50.0)	4 (50.0)	8 (7.3)		
Stomach	0	7 (100.0)	0	7 (6.4)	1.4 (0.5)	0.004
Ovary	0	0	1 (100.0)	1 (0.9)		NA
Uterus	1 (100.0)	0	0	1 (0.9)		NA
Mediastinum	1 (100.0)	0	0	1 (0.9)		NA
Bladder	0	1 (100.0)	0	1 (0.9)		NA
Mesentery	0	1 (100.0)	0	1 (0.9)		NA

Percents for each row represent row percents except for the “All patients” column.

^a^p-value calculated using the t-test statistic comparing each location versus all other locations. For example, mean PAM stain score comparing lung versus not lung p-value = 0.64.

NA: not applicable.

### PAM immunoreactivity is higher in lower grade NEN but not associated with tumor size or stage

Overall, 64% of tumors were grade 1, 7% were grade 2 and 12% were grade 3 (Table 1). Tumor grade was not available for 17% of tumors. We assessed whether PAM reactivity differed by tumor grade. PAM-positive tumors tended to have a lower grade while PAM-negative tumors tended to have a higher grade (p < 0.001). Although PAM staining is inversely correlated with proliferative activity, the size of PAM-negative (3.1 ± 0.8 cm), and PAM-low (2.8 ± 2.0 cm) and PAM-positive (2.3 ± 2.0 cm) tumors at the time of resection was not different (p = 0.09). Furthermore, PAM staining was similar by NEN stage at the time of tumor resection (Table 1).

### PAM-negative staining is associated with increased risk of death

**Figure 2:**
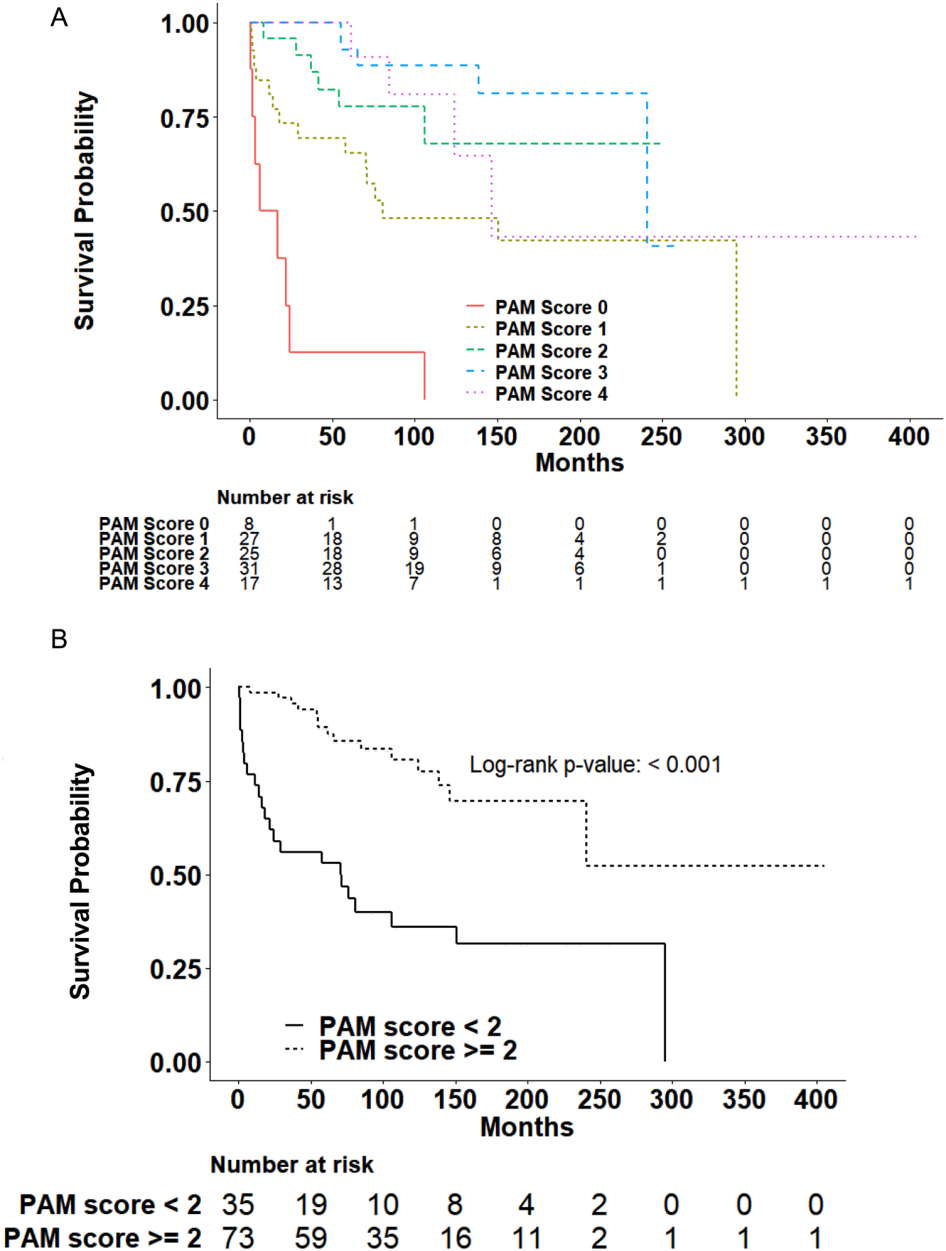
Patient survival analysis according to intensity of peptidylglycine α-amidating monooxygenase (PAM) immunohistochemical reactivity in primary neuroendocrine neoplasms. (**A**) Kaplan–Meier curves were generated to visualize the relationship between PAM staining intensity and survival for all PAM immunoreactivity categories (log-rank p-value < 0.001). (**B**) Kaplan–Meier log rank test was calculated to assess the relationship between PAM staining intensity and survival for patients with tumors with PAM reactivity (< 2) and (≥ 2). Patients with reduced PAM reactivity had a significantly increased risk of early death (p-value < 0.001). Statistical analyses were performed using SAS Version 9.4 (SAS Institute, Cary, NC) and R.

Of the 109 patients that were analyzed, 38 (35%) were deceased. Lower PAM immunoreactivity was significantly associated with patient death (p < 0.001; Table 1). Death occurred in 100% of PAM-negative patients, 55.6% of PAM-low patients and 20.3% of PAM-high individuals. In patients who died, the median time to death in patients with PAM-negative NENs was 11.3 months (IQR 2.4–23.1) compared to 29.4 (IQR 4.1–76.2) and 61.7 (IQR 41.8–124.2) for PAM-low and PAM-high NENs, respectively (Table 1). Interestingly, among patients who died, the intensity of PAM immunoreactivity appeared to directly correlate with the median time to death (score = 0, 11.3 months; score = 1, 29.4 months; score = 2, 39.4 months; score = 3, 65.6 months and score = 4, 104.5 months; Fig. 2A and Supplementary Table S2). There was an increased risk of death among patients who were PAM-negative [HR 11.2 (95% CI 4.9–25.7, p < 0.01)] or PAM-negative and PAM-low [HR 4.1 (95% CI 2.2–7.9, p < 0.01)] compared with other patients (Table 3, Fig. 2B and Supplementary Table S3). Notably, an increased risk of death among PAM-negative patients persisted when individual anatomic sites were excluded from analysis (Supplementary Table S4), suggesting the observed relationship between reduced PAM staining and risk of death was not sensitive to any specific site.

**Table 3:** Cox proportional hazards for death: univariate, and adjusted for disease stage or WHO grade.

	**PAM < 1**	**PAM < 2**	**PAM < 3**
Univariable			
HR (95% confidence interval)	11.20 (4.87–25.72)	4.14 (2.15–7.96)	3.31 (1.57–7.00)
p-value	< 0.0001	< 0.0001	0.002
Adjusted for WHO grade			
HR (95% confidence interval)	1.00 (0.32–3.09)	1.77 (0.72–4.31)	1.96 (0.80–4.82)
p-value	1.0	0.21	0.14
Adjusted for stage of disease			
HR (95% confidence interval)	13.76 (4.16–45.50)	3.49 (1.67–7.26)	3.69 (1.65–8.23)
p-value	< 0.0001	0.0008	0.001

Reference group is PAM-positive for each Cox regression.

**Figure 3:**
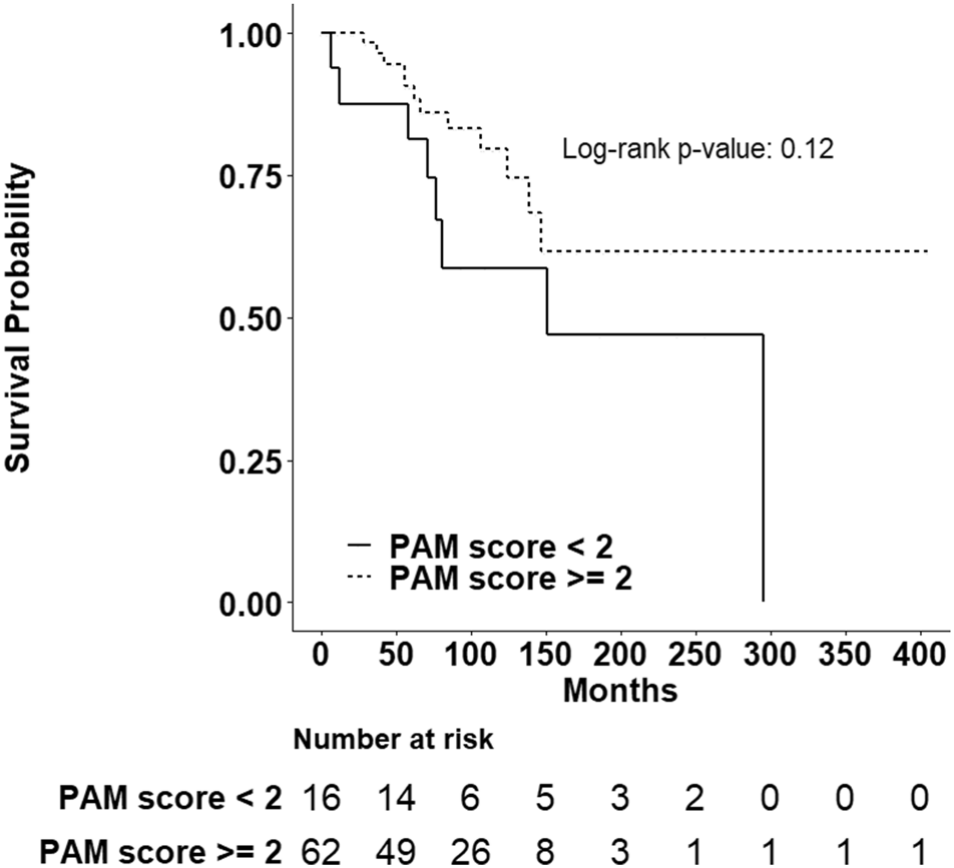
Patient survival analysis according to intensity of peptidylglycine α-amidating monooxygenase (PAM) immunohistochemical reactivity in G1 and G2 primary neuroendocrine neoplasms only. Kaplan–Meier log rank test was calculated to assess the relationship between PAM staining intensity and survival for patients with G1 or G2 tumors only with PAM reactivity (< 2) and (≥ 2). Patients with reduced PAM reactivity did not have a significantly increased risk of early death (p-value = 0.12). Statistical analyses were performed using SAS Version 9.4 (SAS Institute, Cary, NC) and R.

WHO tumor grade and AJCC stage are the best-established predictors of patient survival^2^. Consistent with this, risk of death increased with WHO grade in our cohort [p < 0.001; grade 2, HR 1.48 (0.34–6.44) and grade 3, HR 16.47 (7.45–36.43)] and stage [p = 0.003; stage 2, HR 2.37 (0.69–8.20); stage 3, HR 4.58 (1.45–14.47) and stage 4, HR 6.10 (2.23–16.66)]. Therefore, we assessed whether PAM-negative staining was independently associated with death after adjusting for WHO stage or grade. After adjusting for disease stage, PAM-negative patients continued to have a higher risk of death compared to PAM-positive patients [HR 13.76 (4.16–45.50)]; the association of PAM reactivity with patient death was similar but with smaller hazard ratios after increasing the threshold for determining PAM reactivity (score ≥ 2 or ≥ 3; Table 3). By contrast, the risk of death in PAM-negative (score = 0) patients compared to PAM-positive patients (score ≥ 1) was not significantly increased after adjusting for WHO grade [HR 1.0 (0.32–3.09)]. Interestingly, increasing the threshold for determining PAM-reactivity demonstrated a non-significant trend towards grade-independent risk of death [Table 3: score < 2 h 1.77 (p = 0.21); score < 3 h 1.96 (p = 0.14)]. Hence, PAM staining intensity was not a grade-independent predictor of death. However, this conclusion could reflect the high death rate (100%) among grade 3 NEN patients. Therefore, we examined whether reduced PAM staining intensity (< 2) might identify grade 1 and 2 NEN patients who were more likely to die. Among grade 1 and 2 NEN individuals, 8 of 16 patients (50.0%) with reduced PAM immunostaining died while 12 of 62 patients (19.4%) with PAM-high staining died (Supplementary Table S1). Although reduced PAM immunoreactivity (score < 2) did not statistically predict survival (Fig. 3, p = 0.12), the robustness of this analysis was impaired by limited patient follow-up. In summary, reduced PAM staining intensity was found to be a stage- but not grade-independent predictor of death that might identify grade 1 and 2 NEN patients at highest risk for death.

## Discussion

To optimally manage patients with NENs, reliable distinction between high- and low-risk disease is critical. While current practice, including assessment of WHO grade and tumor stage, are useful for guiding patient prognosis, they lack sufficient accuracy to predict individual tumor behavior^34^. Herein, we identify reduced PAM immunoreactivity of primary NENs as a predictor of reduced survival. While most (potential) prognostic NEN biomarkers are applicable to a subset of NENs, e.g. pancreatic or lung NENs, reduced PAM staining demonstrated utility across a spectrum of NENs^35^. Particularly notable findings of this study were: (A) PAM scoring was highly consistent among three independent reviews; (B) reduced PAM staining was associated with a stage-independent increased risk of death and shorter survival duration among patients who died; and (C) reduced PAM immunoreactivity may identify grade 1 or grade 2 NENs in patients with an increased risk of dying. Given the high variability in clinical disease progression among patients diagnosed at an advanced stage disease and/or with grade 1 or 2 disease, PAM immunostaining may provide a method for identifying tumors at highest risk for progression and thereby yield critical prognostic information.

Predicting the prognosis of well-differentiated NENs remains a challenge. A possible explanation for the variable behavior is that some apparently well-differentiated NENs have undergone de-differentiation toward a more progenitor-like state. For instance, apparently well-differentiated panNENs, which do not exhibit robust staining for the well-differentiated neuroendocrine cell markers chromogranin A, synaptophysin and neural cell adhesion molecule (NCAM), are associated with reduced survival duration^36^. Additionally, in panNENs, reduced expression of PDX1, a differentiated pancreatic β-cell transcription factor, and expression of ARX, found in both alpha-cells and immature hormone-negative proliferative cells, portends stage-independent reduced survival^37,38^. Furthermore, inappropriate expression of cytokeratin 19 (CK19) and/or KIT, both expressed by islet progenitor cells during development but silenced in mature neuroendocrine cells^39–41^, are poor prognostic markers for panNENs^42–45^. Our finding that retained staining for PAM, an enzyme critical to normal peptide hormone bio-activation, indicates a good prognosis supports our hypothesis that so-called well-differentiated NENs are a heterogeneous group of cells with variable degrees of differentiation. Accordingly, we propose that reduced expression of additional proteins involved in differentiated neuroendocrine cell function (biosynthesis and metabolism of bioactive amine and peptide hormones) that are variably expressed by well-differentiated NENs could provide prognostic value. For instance, the clinical significance of L-aromatic amino acid decarboxylase (AADC)^46^, endopeptidase (prohormone convertase 1 and 3, PC1/3) and carboxypeptidase (carboxypeptidase H and E) expression has unknown prognostic implications^47^. While absence of mature neuroendocrine cell markers or presence of progenitor/immature neuroendocrine cell markers could represent de-differentiation of NEN cells towards a more progenitor-like state, we cannot exclude the possibility that PAM-negative tumors arise from a distinct cell population.

Currently, WHO grade 3 NENs are divided into well-differentiated and poorly differentiated NENs. Although this distinction provides important prognostic and treatment sensitivity information, the criteria for distinction are equivocal^48^. The basis for determining a NEN as poorly differentiated are based upon the pathologist impression of pleomorphic cellular nuclei and morphology. We propose that PAM-negative staining could provide a useful distinguishing feature for this determination. Studies evaluating this hypothesis are ongoing.

The retrospective design, small sample size and incomplete patient data of this study could limit the generalizability and reproducibility of this study. Notably, our primary cohort included only 8 WHO grade 2 NENs. The limited representation of grade 2 tumors combined with the poor survival of patients with grade 3 tumors, 92% of which exhibited reduced PAM staining, limited our ability to demonstrate an association between reduced PAM staining and an increased risk of death that was independent of tumor grade. However, it is likely that PAM staining intensity does provide grade-independent prognostic information. For instance, among grade 1 and 2 NENs, we found that 50% of patients with reduced PAM expression died compared to 19.4% of patients with high PAM expression. Hence, our analysis suggests that robust PAM staining indicates a better prognosis while reduced PAM staining indicates a worse prognosis, independent of NEN stage and, potentially, grade and anatomic origin.

In conclusion, there is an unmet need for prognostic biomarkers for patients with NENs. To date, effective biomarker development has been elusive and is generally restricted to specific primary tumor sites of origin. This retrospective study suggests that PAM immunoreactivity provides useful prognostic information across the spectrum of primary tumor sites. Although our findings need to be validated in larger and, ideally, prospective studies, they are provocative and have important potential clinical implications. To date, WHO grade has been the primary variable used to place patients into one of two treatment groups: low grade (WHO grades 1 and 2) and high grade NENs (WHO grade 3). Typically, agents such as somatostatin analogues, everolimus, sunitinib, and ^177^Lu-Dotatate are used for low grade NENs and platinum-based cytotoxic chemotherapy is used for high grade NENs. However, these categories provide imperfect prognostic information. In particular, some patients with grades 1 or 2 NENs have a more rapidly aggressive/progressive disease course and could benefit from early cytotoxic chemotherapy, and some patients with grade 3 NENs have a more indolent course and could benefit from treatments usually reserved for low grade NENs. We need better prognostic biomarkers to optimize treatment selection in NENs and are optimistic that PAM will prove useful for making this determination.

## Methods

### Study cohort

Primary neuroendocrine neoplasms, obtained from the Stanford Tumor Bank or the institutions of referring providers from 1992–2013, were included in a tissue microarray. Tumors were stored for 4–26 years at room temperature. Analysis was restricted to the 109 primary tumors in the array for which there was a clinical pathology report from a Stanford University Hospital pathologist, and for which there was sufficient material. No tumors meeting these criteria were excluded. All tumors were determined to be of neuroendocrine origin based upon appearance on hematoxylin and eosin staining and chromogranin A and/or synaptophysin immunostaining^7^. Primary tumors, which occurred at various anatomic locations including small bowel, pancreas, lung, stomach, large bowel, bladder, uterus, mediastinum, mesentery and ovary, were formalin fixed at the time of removal. A representative tumor paraffin-embedded block from each surgical resection specimen was sampled using 1 mm diameter core punches, with up to 3 cores per specimen if enough tissue was available. The tissue cores were assembled in a paraffin-embedded microarray^49^. Unstained slides of the microarray were made using four-micrometer sections.

### Immunohistochemistry

Following removal of paraffin by washing successively with xylene, 100% ethanol, and 95% (v/v) ethanol, slides were rinsed with deionized water and antigen retrieved in 0.01 mol/L sodium citrate pH 6.0 for 10 min. After cooling, slides were blocked in PBT (5% (v/v) donkey serum (Jackson ImmunoResearch Laboratories, West Grove USA), 0.3% (v/v) Triton- × 100 in Phosphate-Buffered Saline), and an anti-PAM antibody (R&D Systems, Inc. Minneapolis USA; #AF4837, RRID:AB_2158894) was applied at 1:200 (v:v) in PBT, and the slide incubated 12–16 h in a humidified chamber at 4 °C. Secondary antibody (Anti-goat IgG Peroxidase-conjugate, Jackson ImmunoResearch Laboratories, West Grove USA #705–035-147, RRID:AB_2313587) was applied at 1:300 (v:v). Following incubation for 2 h, 3,3′-Diaminobenzidine (DAB) Substrate (Vector Laboratories, Burlingame USA) was prepared and applied for 3 min. The samples were counterstained with hematoxylin for 15 s and imaged.

### Image analysis

Digital images of stained tissue microarray slides were acquired using a fluorescence microscope (BZ-X710, Keyence, Osaka, Japan). Images were white balanced to standardize grading across parallel trials, saved as TIFF files and visualized using Adobe Photoshop (San Jose, CA, USA). All tumor images were independently reviewed by three investigators (TMH, AVV and JPA) and scored from 0–4 based upon the strength of PAM staining (representative scoring in Fig. 1). Reviewers were blinded to all pathologic and clinical data. Scores were recorded along the following criteria: 0, no DAB staining present in any tissue; 1, very faint and scarce DAB staining; 2, staining easily visible in at least 50% of tissue; 3, moderately dark and intense staining across 50% of tissue; 4, very intense dark staining across at least 50% of tissue. Human pancreatic islets were used as a positive control (scored 4) and placenta tissue was used as a negative control (scored 0) across all slides. A sample’s score was recorded as the median of three cores. The median score calculated from all three reviewers was used for analysis (individual reviewer scores are provided in Supplementary Table S1).

### Clinical data

The Stanford Cancer Institute Research Database derives data from all electronic Stanford resources including the electronic medical record (EMR), Cancer Registry, and the pathology reporting system. Date of death was determined by the social security death index and date last known alive was determined by date of last encounter in the EMR as used for censoring. Data were manually curated by a database manager for accuracy and completeness. Tumor grade was determined using current Ki-67 thresholds into Grade 1 (< 3% Ki-67), Grade 2 (3–20% Ki-67) or Grade 3 (> 20% Ki-67)^7^. Well-differentiated and poorly-differentiated Grade 3 tumors were not distinguished. The primary source of Ki-67 values was from central re-evaluation (71 (65%)); however, clinically determined tumor grade from the medical record was used if central re-evaluation Ki-67 values were not available (39 (35%)). Tumor size was obtained from clinical pathology reports. Tumor stage was determined first from the Stanford Cancer Registry. If not available in the curated registry, stage was determined from the pathology report and preoperative radiographic imaging. Tumors were categorized as functional or nonfunctional via chart review, including documentation in clinical notes and laboratory value corroboration.

### Statistics

Descriptive statistics were calculated to compare the distribution of patient characteristics by PAM expression group (negative, 0; low, 1; or high, 2 to 4, defined using the median score from all three reviewers). Categorical variables were compared using the Fisher’s exact test; continuous variables were compared using the Kruskall–Wallis test. To determine the inter-rater reliability for the median score, Krippendorff’s alpha^50^ was calculated with bootstrapped estimates of the confidence interval. Values of this statistic range from 0 to 1; a 0 value reflects perfect disagreement and a value of 1 reflects perfect agreement. PAM staining intensity by anatomic location of the primary NEN was also assessed using the three PAM expression groups. In addition, the mean PAM stain score for each anatomic location was compared to the mean PAM score of all other PAM locations using the t-test statistic. To evaluate risk of death by PAM expression group, we calculated the time to death from diagnosis. Patients that were alive at the last follow-up visit were censored at that date. We created Kaplan–Meier curves and calculated the log-rank statistic to compare survival by PAM staining score and by PAM expression group. To explore the association of PAM expression and survival, we conducted Cox proportional hazards regression analyses: bivariate and adjusted for stage of disease and WHO grade (separately). We conducted two sensitivity analyses for comparing survival by changing the thresholds for defining PAM reactivity. As exploratory analysis, we conducted these Cox proportional hazards regression analyses separately for the PAM scores obtained from each reviewer. As additional exploratory analysis, to evaluate the sensitivity of our findings to tumor site, we conducted bivariate Cox regression analyses to assess the association between PAM scores (< 1) and risk of death by sequentially excluding each tumor site. Significance testing was conducted at a two-sided alpha of 0.05 without adjustment for multiple comparisons. Analyses were conducted using SAS Version 9.4 (SAS Institute, Cary, NC) and R^51,52^.

### Study approval

This study was approved by and conducted in accordance with relevant guidelines of the Institutional Review Board of Stanford University. Prior to inclusion in the study, the Stanford Tissue Bank obtained written informed consent for storage and study of discarded tumor tissue.

## Supplementary information

Supplementary file1 (PDF 690 kb)
