# Supplementary material for: PAM staining intensity of primary neuroendocrine neoplasms is a potential prognostic biomarker
Source: Sci Rep. 2020 Jul 2;10:10943. doi: 10.1038/s41598-020-68071-6 (PMC7331689; doi:10.1038/s41598-020-68071-6)
Supplement: Supplementary file 1 — Supplementary file1 (PDF 690 kb) [file 41598_2020_68071_MOESM1_ESM.pdf]

Supplementary Data for:

PAM Staining Intensity of Primary Neuroendocrine Neoplasms Is A Potential Prognostic Biomarker

Timothy M. Horton, Vandana Sundaram, Christine Hye-Jin Lee, Kathleen Hornbacker, Aidan Van Vleck, Kaisha N. Benjamin, Allison Zemek, Teri A. Longacre, Pamela L. Kunz, Justin P. Annes*

Table of Contents

Supplementary Table S1. NEN cohort information and PAM immunoreactivity scoring **S2**

Supplementary Table S2. Characteristics of study population by PAM median stain. **S4**

Supplementary Table S3. Cox proportional hazards regressions for death: univariate and adjusted for disease stage or WHO grade: Graders 1-3. **S5**

Supplementary Table S4. Cox proportional hazards for death: univariate: excluding one site at a time. **S6**

Supplementary Table S1. NEN cohort information and PAM immunoreactivity scoring

Subject	Age at diagnosis	Sex	Site	Functional Status	WHO grade	Tumor size (cm)	Stage at resection	Died	Length of followup (months)	Median PAM score	Grader1 Median Pam Score	Grader2 Median Pam Score	Grader3 Median Pam Score
1	31	F	Appendix	Non-functional	Unknown	0.2	2	No	1.533	1	1	1	1
2	37	M	Appendix	Non-functional	2	1.2	2	No	61.5	2	2	1	2
3	79	M	Appendix	Unknown	1	0.4	Unknown	No	48.233	3	3	3	2
4	73	M	Bladder	Non-functional	3	2.2	2	Yes	29.4	1	1	1	1
5	53	M	Bronchus	Unknown	Unknown	.	Unknown	No	248.733	2	1	2	2
6	54	F	Bronchus	Unknown	Unknown	.	Unknown	No	218.1	2	2	2	2
7	56	F	Colon	Non-functional	3	4	3	Yes	14.033	1	2	1	1
8	88	F	Colon	Non-functional	3	6	4	Yes	1.067	1	1	1	1
9	59	M	Colon	Unknown	1	.	1	Yes	5.967	0	0	0	1
10	74	F	Colon	Non-functional	3	4	3	Yes	3.4	0	0	0	0
11	40	F	Colon	Non-functional	1	3.8	3	No	108.8	2	2	3	2
12	58	F	Colon	Functional	1	.	4	Yes	150.867	1	2	1	1
13	49	F	Colon	Non-functional	1	0.6	1	No	0	2	2	2	3
14	54	F	Colon	Non-functional	1	1.5	3	No	115.033	4	4	4	4
15	82	M	Duodenom	Unknown	1	.	Unknown	Yes	76.2	1	0	1	1
16	69	M	Duodenom	Non-functional	1	0.7	2	No	3.9	2	2	2	3
17	66	F	Duodenom	Unknown	3	.	1	Yes	1.567	1	1	0	1
18	69	M	Duodenom	Non-functional	1	2	2	Yes	61.667	4	4	4	4
19	59	F	Duodenom	Non-functional	1	1.3	2	No	94.933	4	4	4	3
20	54	M	Ileum	Non-functional	1	1.5	3	No	90.467	2	2	2	2
21	82	M	Ileum	Non-functional	1	2.5	3	Yes	27.933	2	1	2	3
22	66	F	Ileum	Non-functional	1	3.5	3	No	65.233	3	3	3	3
23	51	M	Ileum	Functional	1	1.5	4	No	126	3	3	3	3
24	65	M	Ileum	Non-functional	1	2.1	3	No	160.3	3	3	3	3
25	75	M	Ileum	Non-functional	1	1.7	4	No	58.667	3	3	3	3
26	54	M	Ileum	Non-functional	1	6.6	4	Yes	57.967	1	1	1	2
27	85	F	Ileum	Non-functional	1	1.3	4	Yes	124.2	4	4	4	4
28	48	M	Ileum	Unknown	1	1.1	4	Yes	55.067	3	3	3	3
29	65	M	Ileum	Functional	1	0.9	4	Yes	55.2	3	2	4	3
30	55	M	Ileum	Functional	1	.	4	Yes	84.767	4	4	4	4
31	62	M	Ileum	Non-functional	1	2.2	3	No	103.267	4	4	4	3
32	50	F	Ileum	Functional	1	1.7	3	No	59.433	4	4	4	3
33	51	F	Jejunum	Functional	1	2.5	4	No	256.533	1	1	1	1
34	78	F	Lung lobe	Non-functional	Unknown	1.6	2	Yes	54.2	2	3	2	2
35	55	M	Lung lobe	Non-functional	3	2.9	3	Yes	106.233	0	0	0	0
36	72	F	Lung lobe	Non-functional	3	2.5	4	Yes	8.4	2	2	1	2
37	65	M	Lung lobe	Non-functional	3	2.5	1	Yes	24.333	0	0	0	0
38	61	F	Lung lobe	Non-functional	Unknown	3	4	Yes	2.767	1	1	1	1
39	60	F	Lung lobe	Non-functional	Unknown	.	Unknown	Yes	21.867	0	1	0	0
40	75	M	Lung lobe	Non-functional	3	1	1	Yes	71.233	1	1	1	1
41	66	F	Lung lobe	Unknown	Unknown	.	Unknown	Yes	16.6	0	0	0	0
42	66	F	Lung lobe	Unknown	Unknown	.	Unknown	No	227.5	1	1	1	1
43	50	F	Lung lobe	Unknown	Unknown	.	Unknown	No	217.267	2	2	2	1
44	58	F	Lung lobe	Unknown	Unknown	.	1	No	195.767	1	1	2	1
45	82	M	Lung lobe	Unknown	3	6	4	Yes	4.133	1	1	2	1
46	55	F	Lung lobe	Unknown	Unknown	.	Unknown	No	226.333	2	2	2	2
47	78	F	Lung lobe	Non-functional	1	1.5	1	No	101.267	2	3	2	2
48	61	F	Lung lobe	Non-functional	1	1	1	No	66.367	3	3	3	3
49	63	F	Lung lobe	Non-functional	1	3.3	1	No	143.033	3	3	3	2
50	52	F	Lung lobe	Non-functional	1	1.6	1	No	198.133	2	2	2	2
51	76	F	Lung lobe	Unknown	Unknown	.	1	No	233.533	3	3	3	3
52	59	F	Lung lobe	Non-functional	Unknown	1.4	2	No	139.133	3	3	4	3
53	50	F	Lung lobe	Non-functional	Unknown	1.5	Unknown	No	201.9	3	3	3	3
54	71	F	Lung lobe	Functional	Unknown	.	Unknown	Yes	240.8	3	2	3	3
55	42	F	Lung lobe	Functional	2	9	2	No	211.7	3	4	3	3
56	44	M	Lung lobe	Non-functional	Unknown	1.6	1	No	259.933	3	3	3	3
57	68	F	Lung lobe	Non-functional	1	1.4	1	No	83.267	4	4	3	4
58	43	M	Lung lobe	Non-functional	1	3.5	1	No	95.633	3	2	3	3
59	72	F	Lung lobe	Non-functional	2	2.1	1	No	40.267	4	4	4	4
60	84	F	Lung lobe	Non-functional	1	2	1	No	21.7	4	4	4	4
61	43	M	Lung lobe	Unknown	Unknown	.	Unknown	No	.	4	4	4	4
62	66	F	Mesentery	Functional	2	.	4	Yes	41.767	2	2	3	2
63	80	F	Mediastinum	Non-functional	3	.	3	Yes	0.6	0	0	0	0
64	38	F	Ovary	Non-functional	1	9	1	No	56.267	4	4	4	4

Subject	Age at diagnosis	Sex	Site	Functional Status	WHO grade	Tumor size (cm)	Stage at resection	Died	Length of followup (months)	Median PAM score	Grader1 Median Pam Score	Grader2 Median Pam Score	Grader3 Median Pam Score
65	78	F	Pancreas	Non-functional	3	2.8	3	Yes	18.2	1	1	1	1
66	40	M	Pancreas	Non-functional	1	.	2	No	237.533	1	1	1	0
67	50	F	Pancreas	Non-functional	1	.	4	Yes	80.733	1	1	1	2
68	56	F	Pancreas	Non-functional	2	4	1	No	69.5	1	1	1	2
69	55	F	Pancreas	Non-functional	1	0.7	1	No	63.5	2	1	2	2
70	70	M	Pancreas	Non-functional	1	9	2	No	68.6	2	2	2	2
71	62	M	Pancreas	Non-functional	1	1.8	1	No	103.667	3	4	3	3
72	43	F	Pancreas	Non-functional	1	1.2	1	No	119.567	3	3	2	3
73	40	M	Pancreas	Functional	1	7.5	4	No	171.067	3	3	3	2
74	69	M	Pancreas	Non-functional	1	2.6	1	No	75.933	2	2	1	2
75	61	F	Pancreas	Non-functional	1	1.5	1	No	93.2	1	1	1	1
76	44	M	Pancreas	Non-functional	1	1	1	No	82.4	2	2	2	3
77	68	M	Pancreas	Non-functional	1	0.6	1	No	29.8	3	3	3	2
78	65	M	Pancreas	Non-functional	1	7	4	No	60.867	3	2	3	3
79	36	M	Pancreas	Functional	1	.	Unknown	No	405.267	4	4	4	4
80	41	F	Pancreas	Functional	1	0.7	1	No	128.233	4	4	4	4
81	70	F	Pancreas	Functional	1	1.8	2	No	134.8	3	4	3	3
82	74	F	Pancreas	Unknown	1	5	2	No	105.7	3	3	2	3
83	72	M	Pancreas	Non-functional	1	1.5	1	No	107.267	3	4	3	3
84	62	F	Pancreas	Non-functional	1	2	4	No	109.733	1	1	2	1
85	67	M	Pancreas	Functional	1	2	4	Yes	146.433	4	4	4	2
86	35	M	Pancreas	Functional	1	1.4	1	No	158.4	3	4	3	3
87	49	M	Pancreas	Non-functional	1	2.2	1	No	20.233	4	4	4	4
88	60	M	Pancreas	Non-functional	1	1	1	No	36.033	4	4	4	4
89	79	M	Rectal	Non-functional	1	0.5	1	Yes	70.667	1	1	0	1
90	59	F	Rectal	Non-functional	1	0.8	1	No	78.367	1	1	2	1
91	63	F	Rectal	Non-functional	1	0.8	2	Yes	106	2	2	2	3
92	64	F	Rectal	Non-functional	1	0.4	1	No	11.067	3	3	3	3
93	50	F	Rectal	Non-functional	1	0.7	1	No	34.533	2	2	1	2
94	53	F	Rectal	Non-functional	1	.	1	No	233.5	3	3	3	2
95	52	F	Rectal	Non-functional	1	0.3	1	No	60.9	3	3	3	3
96	50	F	Rectal	Non-functional	1	.	1	No	148.733	4	4	3	4
97	70	M	Small bowel	Functional	1	.	4	Yes	37.033	2	2	1	2
98	46	F	Small bowel	Functional	1	1.6	4	No	185.833	1	1	1	2
99	80	M	Small bowel	Functional	1	1.6	2	Yes	65.633	3	3	3	2
100	74	M	Small bowel	Non-functional	1	2.5	3	Yes	138.6	3	3	4	3
101	76	M	Small bowel	Non-functional	1	0.6	2	No	114.1	3	3	3	4
102	66	M	Stomach	Non-functional	2	4.5	4	Yes	11.667	1	1	1	1
103	78	F	Stomach	Non-functional	1	0.15	1	No	188.933	2	3	2	2
104	56	F	Stomach	Unknown	1	.	Unknown	Yes	295.233	1	1	1	0
105	53	M	Stomach	Unknown	Unknown	.	Unknown	No	199.767	1	1	1	1
106	64	F	Stomach	Non-functional	2	0.4	1	No	74.9	1	1	1	1
107	67	M	Stomach	Non-functional	1	1.5	2	No	88.233	2	1	2	2
108	56	F	Stomach	Unknown	2	.	1	No	74.433	2	2	3	2
109	66	F	Uterus	Unknown	3	.	4	Yes	1.433	0	0	0	0

Supplementary Table S2. Characteristics of study population by PAM median stain.

Characteristic	PAM Expression Group									
	Median stain 0		Median stain 1		Median stain 2		Median stain 3		Median stain 4	
Total	8 (7%)		27 (25%)		25 (23%)		31 (28%)		18 (17%)	
Age at diagnosis, years; Median (IQR)	66 (60-70)		61 (54-73)		56.0 (52-73)		64 (50-72)		57 (49-68)	
Sex, n (%)										
F	5	62.5	18	66.7	15	60.0	14	45.2	10	55.6
M	3	37.5	9	33.3	10	40.0	17	54.8	8	44.4
Site, n (%)										
Lung	4	50.0	5	18.5	8	32.0	9	29.0	4	22.2
Pancreas	0		6	22.2	4	16.0	9	29.0	5	27.8
Small bowel	0		5	18.5	4	16.0	9	29.0	6	33.3
Large bowel	2	25.0	6	22.2	5	20.0	4	12.9	2	11.1
Stomach	0		4	14.8	4	16.0	9	29.0	6	33.3
Other [A]	2	25.0	1	3.7	1	4.0	0		1	5.6
Functional status, n (%)										
Functional	0		3	11.1	2	8.0	8	25.8	5	27.8
Non-functional	5	62.5	17	63.0	18	72.0	19	61.3	12	66.7
Unknown	3	37.5	7	25.9	5	20.0	4	12.9	1	5.6
WHO Grade										
1 (Ki67 < 3)	1	12.5	12	44.4	16	64.0	25	80.7	16	88.9
2 (Ki67 3 to 20)	0		3	11.1	3	12.0	1	3.2	1	5.6
3 (Ki67 > 20)	5	62.5	7	25.9	1	4.0	0		0	
Unknown	2	25.0	5	18.5	5	20.0	5	16.1	1	5.6
Tumor size (cm, mean (SD))	3.1 (0.8); n=3		2.8 (2.0); n=18		1.9 (2.0); n=18		2.3 (2.2); n=28		2.2 (2.0); n=14	
Stage at resection, n (%)										
Unknown	2	25.0	4	14.8	4	16.0	3	9.7	2	11.1
1	2	25.0	8	29.6	9	36.0	13	14.9	8	44.4
2	0		3	11.1	6	24.0	6	19.4	2	11.1
3	3	37.5	2	7.4	3	12.0	3	9.7	3	16.7
4	1	12.5	10	37.0	3	12.0	6	19.4	3	16.7
Died	8	100.0	15	55.6	6	24.0	5	16.1	4	22.2
Time to death (months); Median (IQR)	11.3 (2.4-23.1)		29.4 (4.1-76.2)		39.4 (27.9-54.2)		65.6 (55.2-138.6)		104.5 (73.2-135.3)	

[A] Other sites include the mediastinum, mesentery, bladder, ovary, and uterus.

Supplementary Table S3. Cox proportional hazards regressions for death: univariate and adjusted for disease stage or WHO grade

Supplementary Table S3A. Cox proportional hazards regressions for death: univariate and adjusted for disease stage or WHO grade: Grader 1.

	PAM < 1	PAM < 2	PAM < 3
Univariable			
HR (95% confidence interval),	8.83 (3.92-19.91)	3.32 (1.74-6.35)	3.69 (1.69-8.06)
p-value	<0.001	0.0003	0.001
Adjusted for WHO grade			
HR (95% confidence interval)	1.28 (0.44-3.76)	1.62 (0.69-3.83)	2.58 (1.02-6.54)
p-value	0.66	0.27	0.04
Adjusted for stage of disease			
HR (95% confidence interval)	13.76 (4.16-45.50)	3.24 (1.60-6.53)	3.51 (1.56-7.92)
p-value	<0.0001	0.001	0.002

Note: reference group is PAM-positive for each Cox regression

Supplementary Table S3B. Cox proportional hazards regressions for death: univariate and adjusted for disease stage or WHO grade: Grader 2.

	PAM < 1	PAM < 2	PAM < 3
Univariable			
HR (95% confidence interval)	11.20 (5.18-24.21)	4.92 (2.53-9.56)	2.92 (1.42-6.01)
p-value	<0.0001	<0.0001	0.004
Adjusted for WHO grade			
HR (95% confidence interval)	1.63 (0.55-4.80)	2.11 (0.87-5.14)	1.65 (0.68-4.00)
p-value	0.38	0.10	0.27
Adjusted for stage of disease			
HR (95% confidence interval)	19.50 (6.37-59.68)	4.19 (2.01-8.72)	3.53 (1.62-7.69)
p-value	<0.0001	0.0001	0.002

Note: reference group is PAM-positive for each Cox regression

Supplementary Table S3C. Cox proportional hazards regressions for death: univariate and adjusted for disease stage or WHO grade: Grader 3.

	PAM < 1	PAM < 2	PAM < 3
Univariable			
HR (95% confidence interval)	3.84 (1.71-8.63)	3.79 (2.00-7.21)	2.73 (1.29-5.77)
p-value	0.001	<0.0001	0.009
Adjusted for WHO grade			
HR (95% confidence interval)	0.68 (0.25-1.89)	1.53 (0.58-4.01)	1.41 (0.57-3.47)
p-value	0.46	0.39	0.46
Adjusted for stage of disease			
HR (95% confidence interval)	4.63 (1.56-13.74)	3.93 (1.96-7.91)	2.82 (1.24-6.43)
p-value	0.006	0.0001	0.01

Note: reference group is PAM-positive for each Cox regression

Supplementary Table S4. Cox proportional hazards for death: univariate.

	PAM < 1
All patients<br>HR (95% confidence interval)	11.20 (4.87-25.72)
Excluding large bowel<br>HR (95% confidence interval)	10.42 (4.08-26.61)
Excluding lung	64.86 (11.46-367.10)
Excluding other site	9.21 (3.64-23.34)
Excluding pancreas	9.04 (3.89-21.00)
Excluding small bowel	12.46 (5.05-30.74)
Excluding stomach	11.00 (4.76-25.42)

Note: reference group is PAM-positive for each Cox regression
